# Plant intelligence dux: a comprehensive rebuttal of Kingsland and Taiz

**DOI:** 10.1007/s00709-024-02005-1

**Published:** 2024-11-06

**Authors:** Anthony Trewavas

**Affiliations:** Institute of Molecular Plant Science, Kings Buildings, University of Edinburgh, EH9 3JH Edinburgh, Scotland

**Keywords:** Plant intelligence, Darwin, Learning and memory, Single cell intelligence

## Abstract

Intelligence is a fundamental property for all life enabling an increased probability of survival and reproduction under wild circumstances. Kingsland and Taiz (2024) think that plants are not intelligent but seem unaware of the extensive literature about intelligence, memory, learning and chromatin topology in plants. Their views are consequently rejected. Their claim of fake quotations is shown to result from faulty reasoning and lack of understanding of practical biology. Their use of social media as scholarly evidence is unacceptable. Darwin’s views on intelligence are described, and their pertinence to the adaptive responses of plants is discussed. Justifications for comments I have made concerning McClintock and her “thoughtful” cell, von Sachs writings as indicating purpose (teleonomy) to plant behaviour, Went and Thimann’s allusions to plant intelligence and Bose legacy as the father of plant electrophysiology are described. These scientists were usually first in their field of knowledge, and their understanding was consequently deeper. The article finishes with a brief critical analysis of the 36 scientists who were used to condemn plant neurobiology as of no use. It is concluded that participants signed up to a false prospectus because contrary evidence was omitted.

## Introduction

My contributions to the topic of plant intelligence began in 1999 when I published an invited article entitled “How plants learn” (Trewavas 1999). In 2002, whilst in conversation with one of the editors of Nature, I mentioned I was writing an article on plant intelligence and was invited to provide a very short note, on this issue that duly emerged (Trewavas 2002). In 2003, I published a longer invited article entitled “Aspects of Plant Intelligence” (Trewavas (2003) which elicited some critical reaction (Trewavas 2004; Firn 2004). Currently, Trewavas (2003) has over 670 citations indicating interest.

The primary problem in recognising plant intelligence arises because we ourselves are animals and see the natural world through animal-tinted glasses. Animals usually have to move to find food. The animal timescale is very uniform and makes it easy to casually identify intelligent behaviour. Due to the need to obtain water from the soil, however, evolutionary pressures for the individual plant to move around were absent, giving rise to the impression of plants as still life. Experimental investigations indicated the falsity of that view but it still remains the public assessment. In 1990, my research group developed the aequorin method for detecting changes in cytoplasmic calcium (Knight et al. 1991). The results were a shock; simple signals such as touch, blue light and others instituted changes in less than an estimated tenth of a second. The following transient lasted only some 30 s and was entirely similar, if not identical, to those observed in animals. Animals and plants were not so different after all. The thinking that followed gave rise to the three papers indicated above and to the slow realisation that intelligence was one consequence. In Trewavas (2003), I characterised plant intelligence as adaptively variable behaviour during the lifetime of the individual (Stenhouse 1974). It was relatively easy to show that changes in cytoplasmic calcium were an essential first step in subsequent phenotypic changes. Scherzer et al. (2022) is a recent nice example. Adaptive responses were identified by Dobzhansky (1956) as behaviour increasing the probability of survival. Whatever the subsequent processes were, they had survived from the origin of plants several billion years back in which an amoebic ancestor captured a blue-green alga that became symbiotic and with time changed into a chloroplast.

The identification of plants as intelligent organisms is now widespread and reflects as much a growing awareness of how mankind has destroyed so much of the natural environment. Dignifying plants with intelligence capabilities is just one arm in attempts to increase conservation and return unnecessarily damaged environments to natural circumstances. It was used for the formulation of Swiss law on the respect to be shown to plants and to their dignity. We depend on plants for so many services that this is fast becoming essential. Inevitably negative reactions occurred, and plant intelligence has received its share of criticism, but continued research is indicating the errors in these claims.

## What Kingsland and Taiz claim

According to Kingsland and Taiz (2024), “Proponents of the concepts of plant intelligence…depend heavily on the rhetorical use of historical sources as evidence to argue that eminent past scientists have supported ideas of plant intelligence, memory, learning, decision-making and agency”. Historical sources include writings by Charles Darwin, von Sachs, Went and Thimann, Mcclintock and apparently Lamarck. Kingsland and Taiz (2024) conclude there is a consistent pattern of distortion, including the use of fake quotations and alterations of quotations (apparently leaving out apostrophes with good reason), “a consistent pattern of distortion which suggests confirmation bias”. I reject the whole of their paper and over-dramatised claims without substance. I cannot find the science in it because the overwhelming evidence for plant intelligence has simply been ignored; the above scientists were quoted for other reasons and I do not depend on historical figures. It is easy to make such charges if you do not know the current literature or fail to read the sources claimed to better understand what they do meant Frankly, historians study the past; when dealing with the present, they are often out of their depth. This group centred around Taiz and Robinson never offers constructive criticism, as discussed later, but entirely negative attitudes.

## The actual scientific evidence for plant intelligence, learning and memory

### Nervous systems and brains are not necessary for intelligent behaviour, but neural networks almost certainly are

Primary evidence for bacterial intelligence is to be found in Trewavas (2014. p201-210), and there have subsequently been more recent additions confirming bacterial intelligence and intelligent behavior (Pinto and Mascher 2016; Majumdar and Pal 2017; Westerhoff et al. 2014; Lyon 2015; Koshland 1980). Allmann (1999. p3-8) lists out the brain-like functions in unicellular organisms. Learning, memory, intelligence, decision-making, quorum sensing and self-awareness are all indicated to be present in these and previous articles on bacterial behaviour.

Westerhoff et al. (2014), who argue intelligence from a systems perspective, also include protozoan intelligence. Frances Darwin, son of Charles, stated in 1908 that his father was fascinated by the similarities between the behaviour of the lower animals and plants. Apart from quorum sensing, similar behaviours to bacterial intelligence were identified in protozoa (*Amoeba*, *Paramecium, Stentor* (Jennings 1904, 1906). These organisms, as do plants, respond adaptively to challenging environmental circumstances. Stentor uses trial and error learning in constructing intentional behaviour (Dexter et al. 2019; Tang and Marshall 2018; Jennings 1904, 1906). *Physarum*, a single coenocytic cell, has recently been characterised as behaving intelligently in a variety of circumstances (summarised in Trewavas 2017). Binet (1897. p3), who went on later to construct the first intelligence test to identify children with special educational needs, authored a textbook on protozoan behaviour and concluded that they were intelligent organisms. Jennings (1906 p334) arrived at an identical conclusion. Research on single cell plants, like *Chlamydomonas, *confirms their adaptive abilities to environmental stresses (de Carpentier et al. 2019; Shetty et al. 2019; Sasso et al. 2018) and behavioural changes in response to environmental stress. The plant/animal divide is assumed to have occurred when an amoebic ancestor engulfed a blue-green bacterium which eventually evolved into a chloroplast 2 billion years ago (McFadden et al. 1994). Intelligent behaviour was thus present in the amoebic primordial plant cell. Kingsland and Taiz (2024) have to assume that such behaviour was lost some time later by the evolving plant despite the environmental challenges which needed intelligent responses, particularly in the subsequent advances onto the land.

*Amoeba* (de la Fuente et al. 2019), *Paramecium* (Gershman et al. 2021) and brainless jellyfish (Botton-Amior et al. 2023) also learn from experience and have also demonstrated the abilities, like Pavlov’s dogs, to learn by association. Such abilities indicate that single cells can remember a previous stimulus and then flexibly join it together with another environmentally remote signal. Learning by association, although rarely looked for, is present in plants (Goh et al. 2003; Meyer et al. 2014), and adaptive behaviours are found throughout the plant kingdom. The reason that there are not more examples of learning by association may be that the authors receive unwarranted negative attacks when they do attempt to publish such information. The final stage of learning by association is present in plants; two remote signals, now joined together, has already been referenced (Trewavas 2021).

### Cellular neural networks are likely fundamental to behaviour

Neural networks, a term that originated in artificial neural networks, are now recognised to be present in cells arising from the formation of complexes of numerous interacting proteins whose equivalents are present in all organisms (Bray 2009). Bray (2009) quotes McClintock’s 1984 “thoughtful cell” on his frontpiece (confirmation bias or respect?). Neural networks are discussed in greater detail in Trewavas (2014: chapter 23) and arise more particularly from protein kinase networks. Golden (2004) asks participants in an EMBO conference on neural networks in bacteria to “think like a bacterium”. See the conference summary (Armitage et al. 2005) and Sarkar et al. (2021) to see its sophistication.

One key here may be the almost ubiquitous use of cytoplasmic calcium in plant and single cell learning. *Chlamydomonas*, a single cell plant, for example, has 37 calcium-dependent protein kinases similar in number to the 34 in *Arabidopsis* (Merchant et al. (2007)*.* These can feed into a potential and complex assessment network of over 700 protein kinases in *Chlamydomonas* (Merchant et al. 2007) and over a thousand in *Arabidopsis* (Wang et al. 2020).

### Do Kingsland and Taiz understand what intelligence is? Distinguishing biological intelligence from human intelligence

Throughout their article, Kingsland and Taiz (2024) use the word intelligence without stating what they mean by it. From my experience with the first introduction of plant intelligence in 2003, most plant scientists have, unsurprisingly, no knowledge of the intelligence literature. Given the wealth of present biology knowledge that must be read, this is no surprise. When faced with the word intelligence, they fall back, incorrectly, on their limited experience of human intelligence and IQ which has no relevance to plants. Chamovitz (2018), despite being a very good molecular plant biologist, exemplifies this extremely common error and was corrected in Calvo et al. (2020). Kingsland and Taiz (2024) subscribe to this common error too by claiming Chamovitz (2018) to be right.

The psychologist, Anna Anastasi (1983), distinguishes biological intelligence from the psychologist’s human intelligence. Biological “intelligence is not an entity within the organism but a quality of behaviour. Intelligent behaviour is adaptive insofar as it represents effective ways of meeting the demands of a changing environment. Such behaviour varies with the species and the context in which the individual lives. Intelligence is a pluralist concept”. This is applicable to both plants and animals. Intelligence involves changes in behaviour and the environmental conditions that elicit it. It has been summarised as (organism ↔ environment) (Gilroy and Trewavas 2023).

Behaviour is what plants do: germination, growth, flowering to seed formation and back to germination. Adaptive behaviour leads to modification of this cycle to enable it to continue when circumstances demand. In Beer (1990.preface) “Intelligence as Adaptive Behaviour”, the same quality is identified in animals. “I wish to emphasise instead the more universal ability of animals to cope with the complex dynamic and unpredictable world in which they live. To me this penchant for adaptive behaviour is the essence of intelligence”. Adaptive behaviours in plants are induced during imbalances in water supplies, light, gravity, minerals, predation, wind, stressful temperatures, predation, amongst others (Trewavas 2014) that result in phenotypic and chemotypic changes to improve the probability of survival in wild circumstances (Gilroy and Trewavas 2023). The literature here is enormous.

Anastasi (1983) also clarified the difference with human intelligence. “In the human species the influence of learning on intelligent behaviour has been immensely enhanced through the intergenerational cultural transmission of a rapidly accumulating mountain of knowledge. This influence has been further strengthened through the organised transmission of knowledge by means of formal schooling. Traditional IQ tests simply measure scholastic aptitude or academic intelligence”. The ultimate driving force of life is survival (Calvo et al. 2020). Behaviour that improves survival and its continuation to reproduce is profitable to species survival. Monod (1972) identifies teleonomy as important to these fundamental properties.

### Erroneous assumptions that plants are simple and therefore cannot express intelligent behaviour

Plants should be considered complex adaptive systems because such behaviour is essential in wilderness conditions. But a common assumption is that plants are simple and are not complex. In my view, this notion arises mainly from laboratory experience in which plants can be compelled to behave by choice of imposed environment. Stating that plants are composed of leaves, roots, buds and sometimes flowers is also simplistic when considering the range of forms of angiosperm species. The difficulties and failure of getting reproducible growth and cell number when using inbred seeds and exacting growth and soil conditions (Massonet et al. 2010) only indicate that in the wilderness, the variations are going to be much larger. Development is a first-order Markov cycle (Gilroy and Trewavas 2023) reflecting continual adjustment of developing form as the environment varies. Bazzaz (1996. p112-114) provides diagrams to indicate the enormous degree of phenotypic variation in *Abutilon* when grown in the presence of itself or *Datura*, *Polygonum* or *Setaria*.

A prime example of implying plants as simple organisms comes from the use of Mendel as introducing courses in genetics (Bapty 2023). Mendel’s characters subtly suggest a simplicity in genetics that easily and misleadingly encourages belief in genetic determinism. But Mendel’s genes (there is more than one gene specifying wrinkled seeds (Rayner et al. 2017; Radick 2022) are the exceptions compared to the complex realities of 99% of morphological and physiological characters that do not behave simply. Most traits in plants use multiple interacting gene products.

The observation that Mendel, in his will, requested all his seeds and plants should be destroyed should at least raise a question, when taught. Why did he insist on this when his results were apparently so clear-cut or did later experiments on other races of *Pisum sativum* indicate an apparent failure? Whilst Mendel’s genes have been identified, they seem apparently to be no longer in the same race of *Pisum*. Mendel may simply have been lucky (Rayner et al. 2017). Other plant breeders in the early twentieth century were unable to repeat many of Mendel’s simple observations (Radick 2022). Mendel’s seeds had been domesticated for many years and thus generated a specific race. Enormous numbers of domesticated races can be generated from both plants and animals by animal and plant breeders as indicated by Darwin (1859).

Teaching of Mendel for introductory genetics can quickly lead to the perception that plants are therefore simple too. And if students are taught that all that is needed to explain any of the dozen or so tropic phenomena are marginal redistributions of auxin, then it confirms the apparent simplicity again. Textbooks tend to write about scientific idealisms! Not the reality that composes the natural world.

### Learning and memory are the bases of all kinds of intelligence (Okano et al. 2000)

1. Chromatin topological changes are the basis of some or all plant memories

Kingsland and Taiz (2024) think memories do not exist in plants. Priming was a term initially used to describe the ability of plants to mount enhanced future responses from a first environmental challenge, leading to an adaptive increase in the probability of survival. The stressful situations initially described as priming were induced by chemicals, pathogens and predators. Priming is now recognised to be simply a memory that is more quickly accessed enabling higher responses on subsequent challenge (Kambona et al. 2023). Kambona et al. (2023) list 15 published examples of established memory in plants often the result of what are termed stressful conditions. Another five are referenced in Trewavas (2014. p274). There are more than 60 reported examples of transgenerational inheritance of parental experience of a variety of environmental conditions by siblings (Gilroy and Trewavas 2023). These proactive memories, improving species survival, last anywhere from 1 to 9 generations. Transgenerational inheritance represents another memory (Cao and Chen 2024; Gilroy and Trewavas 2023).

For these transgenerational memories to be formed, it requires, at the minimum, chromatin topological alterations to be applied by the parent cell. Specific chromatin structures are then retained through sexual reproduction. The probable bases of environmental memories are changes in chromatin structure and have been identified as such (Bhadouriya et al. 2021; Barrett and Wood 2008; Pei et al. 2021; Phanstiel and Wang 2022; Harris et al. 2023; Friedrich et al. 2019; Iwasaki and Paszkowski 2014; Gentrya and Hennig 2014). The mechanism of memory is similar between plants and animals (Watson and Tsai 2017). The altered form of chromatin topology takes time to implement but is then already available for subsequent responses to build on. Plants can reprogram their genomes (Cao and Chen 2024; Schapiro 2011), indicating both cells and the parent plant act as agents controlling their own behaviour and proactively those of siblings, something that Kingsland and Taiz do not apparently understand or know about.

Transgenerational inheritance is proactive behaviour on behalf of the species. Plants act as agents (Sultan et al. 2022) able to control their own behaviour and that of siblings. Transgenerational inheritances are analogous to cultural transmission of information in animals.

2. Learning is well-established and is electrical in character

Memory cannot be acquired without learning, and a great deal is now known about the learning process (Trewavas 1999). In molecular characteristics, it shares strong similarity between animals and plants (Gilroy and Trewavas 2023; Luan 2011; Kudla et al. 2018; Dodd et al. 2010). Immediate receipt of almost any environmental signal (at least 20 at the minimum have been established in plants) leads to the initiation of cytoplasmic Ca^2+^ transients via action potentials and other electrical and chemical signals. The kinetic characteristics of the transient are different between different tissues and activated by tissue-specific receptor proteins, again specifically located adjacent to calcium channels. There are 34 genes in *Arabidopsis* expressing calcium-dependent protein kinases that possess substrate discrimination (Curran et al. 2011). Information is passed on to complex protein kinase networks including neural networks (Wang et al. 2020). Shortly after the initial cytoplasmic Ca^2+^ signal (usually cytoplasmic in appearance), nuclear Ca^2+^ increases (Charpentier 2018; van der Luit et al. 1999) and leads to the translocation of some protein kinases into the nucleus (Adachi et al.^2000^), activating enzymes or modifying their synthesis, that lead to the modification of chromatin structure.

Minorsky (2024) points out that it is only very recently (2015) that Taiz and Steiger’s textbook actually mentioned action potentials, over a century after they were identified and characterised in great detail by Bose (1926). Minorsky (2024) also points out that in their brief mention of this topic, the authors of this textbook introduce naïve readers to some serious misconceptions. Was there some block that prevented an earlier mention than 2015 in this textbook?

### What is necessary for a scientific debate?

Debates can get heated and I suggest certain requirements would better enable sensible scientific debates and exchange of ideas. Respect between all participants. An appreciation that no one is the ultimate repository of wisdom. That different scientists have different world pictures of their understanding of plants and behaviour derived initially from training, research and extent of reading of different kinds of literature (e.g. ecology). Some textbook writers can fall into this trap and consider themselves more informed than others, not recognising that the material included in their book has gone through the sieve of their own perceptions and world picture. Textbooks reflect the biases of their authors. Dogmatic statements should be avoided.

## Answering Kingsland and Taiz errors

### It is the idea that is crucial, not the precise form of words used to express it

In my view, Kingsland and Taiz (2024) fail to understand some basic elements of investigative biology. Biology is not physics with defined laws with exacting equations and highly precise circumstances needed for exacting repetition. If there is one critical feature of biology, it is variation, the source material such as a group of individuals of the same species that has to manage with variation between each of them from the other (Watahiki and Trewavas 2019). Statistics can be used to simplify but these can disguise the interesting question; what about the outliers? How do they respond? The mean value also disguises, when it is assumed to have meaning, that nature does not use identical routes; it relies on variation of mechanism and avoids the question of what causes the variation in the first place (Watahiki and Trewavas 2019). The phenomenon is called individuality and is fundamental to biology and the means of explaining it.

When writing a scientific paper (I have written some 300), there is always an introduction that explains the current state of understanding with concise reference to relevant published precedents but in the author’s own words; we do not sit down and seek out the precise form of words used by each preceding publication because it is simply not necessary. The critical point is to convey the idea and if that melds with current understanding that is sufficient.

There must be a hundred ways of explaining the overall process of natural selection; we know of at least two that advanced the notion independently, and Darwin himself, in public or with friends, relatives and other workers, unless he learned it parrot fashion, would have used different means of expression in explanation each time. But each of the hundred will be correct. What Darwin wrote down is just one formulation. None of these are fakes or exaggerations as Kingsland and Taiz (2024) try to make out. Darwin’s written version is just one of them, and he was a scientist open to admitted but important errors (Gilroy and Trewavas 2023). But should I expect a science historian to understand the realities of research life, grants, papers and meetings all of which require explanations of a main idea in words that inevitably will be different? Variation means variation in scientists too, each of whom will explain the same thing in different ways.

There seems to be a group referred to by Kingsland and Taiz (2024) and operating on social media that uses online versions of Darwin books to claim that statements attributed to him either did appear in Darwin’s book or did not. If it does not, it is claimed to be fake, not only meaningless but wrong. To me, they are wasting every scientist’s time. By doing it on social media, they avoid the critical assessment necessary for the worth of their activity. They may say but this is attributed to Darwin by those that use them; who else should they attribute it to? I only quote directly when I cannot think of an easy explanatory alternative.

I have used the statement “Intelligence is based on how efficient a species becomes at doing the things they need to survive”, which, when I saw it, reflected with reasonable accuracy my view of what I had remembered Darwin had described about intelligence. Whether he said it is irrelevant to me. I read the book on earthworms some 25 years ago to which it is applicable and is described later. By mistake, I attributed it to 1871 rather than as it should be 1881. Kingsland and Taiz describe that as fake, and exaggeration, which as indicated above, shows no understanding of practising biology. But I already knew that; two European scientists had already pointed that out to me that the date was wrong. A pity Kingsland and Taiz do not show the same level of scientific courtesy.

### Other claims made by Taiz and Kingsland

I have participated in two “debates” with this small group of highly conservative scientists centred around Taiz and Robinson. I find there are certain characteristics of exaggeration, error and an unnecessary indication of emotion that are revealing about the real reason for the objection to plant neurobiology and intelligence in the first place. Take, for example, the claim by Kingsland and Taiz that arguments for plant intelligence, sentience and consciousness would have been anathema to Julius von Sachs. No one knows, certainly not Taiz and Kingsland, what was anathema to von Sachs, except for the one thing which von Sachs indicated was anathema to him. That will be indicated later.

Again, in quoting MacDougal (1908 p175) by Kingsland and Taiz, “In no instance does the activity of the plant involve choice or decision (spoiler alert; they do. Nick 2023), or anything except the most generalised form of consciousness”. This most generalised consciousness is downgraded by Taiz and Kingsland “to responding to stimuli” with no justification. What Macdougal did say is written in the New York Times (1909) article discussing Frances (son of Charles) Darwin’s statement in 1908 that plants retain a faint trace of consciousness of the kind in mankind. MacDougal (1909 p75) wrote “If the proposal of Woodbridge be accepted, that the coupling up of two forms of perceptions gives rise to consciousness then this faculty may be shared by some plants of which the narcissus, or Chinese lily is an example. The orientation by daffodil blossom depends both on the force of gravity and the direction of illumination”. Integration of signals is indicative of a conscious reaction albeit at a low level. Kingsland and Taiz (2024) have put their own (fake?) words into his mouth.

Moreover, in their article, Kingsland and Taiz state that dead people cannot rebut statements made on their behalf, but they ignore that when discussing McDougal and others now dead, including Barlow, who died 7 years ago. They claim that supposed anthropomorphic statements have sometimes been made by us. Not recognising that what is actually anthropomorphic is frankly in the eye of the beholder and not fact, just opinion. The narrowness of conservative attitudes and claims is invariably defeated by advancing knowledge (Minorsky 2024). It is claimed by Kingsland and Taiz (2024) that plant neurobiology has contributed nothing that could not be dealt with by ordinary technology; Minorsky (2024) exposes the falsity of that view.

### Use of names of well-known scientists to bolster claims of intelligence?

When I use such names of such “luminaries”, it is because such people were usually the first to suggest important aspects which changed perspectives on plant and cell intelligence. It is a scientific courtesy to indicate the contribution of each of these since they were the first in their field to do so. But also in my long experience in science, such scientists have usually spent some considerable time thinking through the consequences of what they have found and putting them down on paper. Every plant scientist needs to understand their insights in order to help them understand such concepts and design better experiments. This is not confirmation bias but simply good scholarship.

### Charles Darwin and intelligence

Kingsland and Taiz profess ignorance of what Darwin actually said about intelligence. The most extensive discussion on intelligence by Darwin comes from his experiments and observations on earthworms. He was the first published source on biological intelligence so far as I could find some 25 years ago. Darwin’s interest in earthworm behaviour started in the 1830s, and he involved both relatives, friends and his son in making the observations (Darwin 1881 and Darwin letters (https://www.darwinproject.ac.uk) (University of Cambridge)). Darwin (1881) noted the typical behaviour of earthworms was to drag leaves (as eventual food) into their burrow and asked himself at the beginning as to what is the most efficient way of getting differently shaped leaves, into the ground when randomly distributed on the surface. If they were pointed, then he considered that the efficient way would be to pull the leaf into the burrow, point-first for leaves of that shape because he would do it that way. He records that about 80–85% of worms drag leaves into their burrows by the pointed part of the leaf. Experiments using differently shaped, triangular-like bits of paper confirmed that conclusion. Descriptions then followed using different types of leaf that came from plants that were not native to England with similar conclusions on leaves of pointed shape. Completely round leaves (lime tree, for example) with a tiny point led only to slight, point-based differences in the removal to the earthworm burrow. How then do worms determine the shape in the first place? He describes how they touch the leaf all around (they do not have eyes) and must clearly remember the shape so they can drag it point first into the burrow. Darwin concludes that worms have some degree of intelligence, it is not chance behaviour.

George Romanes, who Darwin first noticed at Cambridge in 1871, became Darwin’s close associate in 1874. In discussion (Darwin 1881), Romanes pointed out that intelligence can only be safely inferred when we see that an individual profits from its own experience. “Now if worms try to drag leaves into the burrow first in one way or another until they at last succeed, they profit at least in each particular instance by experience” was Darwin’s response (Darwin 1881. p.40). Trial and error.

Darwin handed over a lot of material he had accumulated on intelligence to Romanes who included them in his book “Animal Intelligence” (1883). Romanes included a definition of intelligence (Romanes 1883. p17) of “Intentional adaptability of means to ends”. Darwin’s contributions are indicated throughout the book, and they give some further understanding of Darwin’s understanding of intelligence. In my view, Romanes had a better grasp of biological intelligence than Darwin.

“Intelligence is based on how efficient a species becomes at doing the things they need to survive”, is a statement I have used twice and is, in my view, an accurate summary of Darwin’s understanding of intelligence. It is irrelevant to me whether Darwin actually wrote those exact words. If the statement accurately fits the observations (something we do all the time as indicated earlier), then they are Darwin’s written opinions briefly summarised, so they are refenced to Darwin. “Efficient” covers profiting from experience in the recovery of food for survival. The more efficient, the more gain of food: with plants the physical resources of the environment are the equivalent of “food”. The more efficient, gain more, with a similar outlay of energy.

### Darwin includes plants in his discussion of intelligence

On rereading Darwin’s book for this article, I realised that he did mention plants which I had forgotten. In discussing intelligence Darwin says, “We see how difficult it is to judge whether intelligence comes into play, for even plants might sometimes be thought to be thus directed: for instance, when displaced leaves redirect their upper surfaces towards the light by extremely complicated movement and by the shortest course” (Darwin 1881 p38). This is an adaptive response. Von Hartmann (1875) concerning the self-same plant response concludes “If one sees how many means are here to attain the same end, one will be almost tempted to believe that here dwells a secret intelligence which chooses the most appropriate means for the attainment of the end”. The ability of the leaves of many species to perform this task can improve the probability of survival and is therefore adaptive but only in relevant wild circumstances.

### Barbara McClintock and the “thoughtful cell” concept

The refence to her is the oft-repeated statement “a goal for the future would be to determine the extent of knowledge the cell has of itself and how it uses that knowledge in a ‘thoughtful’ manner when challenged” (McClintock 1984). McClintock spent considerable time in thinking because she introduced transposons when the dogma was that the genome was inviolate. Dogmatic voices tried to see her observations destroyed (Keller 1983. p8-10). Is this familiar? She was even described as mad. Such attitudes are common amongst those who fail to see the value of freedom of thought. Keller (1983) spent a lot of time recording conversations with McClintock who indicated the importance of holding on to an idea despite the difficulties “don’t throw it away many good ideas have gone that way”.

Kingsland and Taiz (2024) state that I sometimes modify McClintock’s statement in two ways. One is the insertion of the word “organism” replacing the word “cell”. That is because I read Keller’s book throughout before writing; Kingsland and Taiz have not. I quote from Keller page 200, “Organism” is for her a code word, not simply a plant or animal. She regards cells as organisms and organisms as cells. Note her commitment to the “oneness” of nature (Keller 1983 p.201).

.Another transgression that I made according to Kingsland and Taiz (2024) is my removal of apostrophes around the word thoughtful. Dennis Bray quotes McClintock’s statement with the apostrophes around the word thoughtful but his interpretation is the same as mine. The title of his book “Wetware a computer in every living cell (2009)” identifies what Keller’s statement means to him and to me. Bray has given me numerous insights into cell function with valuable articles published in Nature and elsewhere. Others concur in understanding. “The cell is like a table in which decision makers debate a question and respond collectively to the information put to them” (Levy et al. 2010). The negative attitude of Kingsland and Taiz has given me nothing and does not progress understanding; it is in fact the reverse. The only reason for the apostrophes is that she recognised that thoughtful was not a common word to use about cells.

Barbra McClintock’s “ Knowledge the cell has of itself” and “thoughtful cell” counter the dogma associated with the modern synthesis of evolution that is fast collapsing under its own contradictions (Ball 2023; Noble 2008; Noble and Noble 2023; Schapiro 2011). Her information indicated that the cell is master of the genome and not as commonly assumed the reverse (Trewavas 2014, chapter 21; Schapiro, 2011). Ball (2023) makes this conclusion very clear too. In McClintock’s view, the cell, not the gene, is the basic element of life. “There’s no such thing as a central dogma into which everything will fit-any mechanism you can think of you will find- even if it is the most bizarre form of thinking behaviour” (Keller 1983. p179).

### Went and Thimann (1937)

Went and Thimann wrote (1937 p151) “In tropic responses plants exhibit a kind of intelligence; their movement is of subsequent advantage to them”. “This sensitivity, not otherwise noticeable in plants, explain why it is in this regard to tropistic responses, the parallelism between plants and animals has been so much stressed” (Went and Thimann 1937). Kingsland and Taiz (2024) omit the second sentence and claim that “the context does not mean they thought plants had a kind of intelligence”. That is wrong. Thimann was a chemist and F. Went disappeared into ecology after this publication. The key words here are “advantage”, that is benefit, profit as indicated by Romanes (1883) in his definition of intelligence. Secondly, is the parallelism between plants and animals in movement. Tropic responses are adaptive responses: Romanes definition is as applicable to plants as it is to animals.

Darwin’s earth worm experiments on intelligence and Romanes definition are routinely included in university lectures and particularly in lectures on soil. Only in wild or even agricultural field circumstances will gravitropism be an advantage; it has no benefit in laboratory experiments. I have always reproduced the Went and Thimann statement above because it is the first time that plant physiologists identified a kind of intelligence in plants.

### Julius von Sachs

Von Sachs was for some 60 years a lonely voice concerning a role for purpose in plant development and behaviour. Sachs (1887) p601) said “All those adaptations in the organism are purposeful which contribute to its maintenance and insure its existence”. What upset von Sachs was not the imagined ramblings of Kingsland and Taiz but the following: “Concerning the point, I should wish to anticipate viz the use of the word purpose which many fanatics of the theory of descent would if possible, banish from the language”. “To the purpose means therefore the same as capable of existence” (1887, p10). Purposeful behaviour has the same meaning as intentional behaviour forming part of Romanes (1883) definition of intelligence. Purpose describes goal-directed behaviour. But the use of purpose was condemned by the Modern Synthesis of Evolution that insisted incorrectly that mutations were randomly distributed through the genome. It is now known they are not randomly distributed in bacteria, plants and animals (Martincorena et al. 2012; Zamai 2020: Grey-Monroe et al. 2022). The recovery of purpose, that is, goal direction, in plants is to be found in Russell (1946). He carefully skirts the problems of the dogma of the Modern Synthesis of Evolution.

The Modern Synthesis of Evolution is fast disappearing as it is known that cells control their own genomes (Schapiro 2011). The genome is just another cell organ or organelle. Such purposeful behaviour is now described as teleonomic, and von Sachs had the foresight to appreciate that (Gilroy and Trewavas 2023). If further information on teleonomy is required, then the 15 papers on teleonomy (Biological Journal of the Linnean Society 139: pp341-587) are required reading. Gilroy and Trewavas (2023) on plant teleonomy illustrate how Darwin’s self-admitted error of underestimating environmental influences obviously and particularly on plant evolution, resuscitates purpose and directiveness in plant development and evolution through environmental change.

What is meant by teleonomy or goal-directed behaviour? It can be said that a seed germinates and makes a new plant, or more accurately that a seed germinates to make a new plant. A flower opens to attract pollinators; a fruit ripens to attract predators that will spread its seed. These statements indicate purpose; Von Sachs would have approved.

### JC Bose

Bose is considered justifiably the father of plant electrophysiology with 7–8 books, numerous papers and thousands of experiments on numerous plants. In the preface to his “Nervous mechanism of Plants” (1926), he states “I next tried to find whether ordinary plants, meaning those regarded as insensitive, exhibit characteristic electric responses already known in sensitive plants. I was able to show that every plant is excitable and responds to a stimulus by electric responses of galvanometric negativity the response being abolished at the death of a plant”. In page 157 onwards, he demonstrates that the pulvinus operates electrically under asymmetric light stimulation; this is the tissue that Darwin and von Hartmann referred to earlier in reference to intelligence.

I published several of his results in Calvo et al. (2016) illustrating fatigue and were able to demonstrate a similar fatigue in intracellular calcium during repetitive wind signalling (Knight et al. 1992). What Bose was detecting is undoubtedly interpreted through elevations of cytosolic calcium.

### Lamarck

He was much and incorrectly maligned by proponents of the Modern synthesis. I read through the translation by Packard and isolated what he said of value to plant behaviour. I have published the list twice in Trewavas (2014, 2023). Gilroy and Trewavas (2023) explain his use in understanding plant evolution.

## Errors of confirmation bias apply to Kingsland and Taiz (2024)

The suppression of uncomfortable ideas may be common in religion or in politics, but it is not the path to knowledge and there is no place for it in the endeavour of science.—Carl Sagan (1981) 

I have used this quote from Carl Sagan, husband of Lynn Margulis, and a noted astrophysicist. I am not an astrophysicist but this quote from a television programme in 1980 (Cosmos; Heaven and Hell) summarises my reaction to Alpi et al. (2007). Negative attitudes do not contribute to the path of knowledge, and this group that organised Alpi et al. (2007) seems incapable of constructive criticism but only condemnation.

Much is made by Kingsland and Taiz (2024) of the 36 scientists who signed up to a letter sent around by David Robinson and finally published as Alpi et al. (2007). That claim was that plant neurobiology was of no use and as Minorsky (2024) puts it, an attempt to strangle it at birth. But a counterbalancing statement was not offered as it should have been. The contribution by Alpi et al. (2007) was constructed in my view as an alarmist statement as though plant biology would collapse if people did not sign up. An alternative point of view was not solicited as good scientists would have done.

In 2005/2006, I was contacted by a German scientist, a future signatory of Alpi et al. (2007), asking me to do something about plant neurobiology. The correspondent said I was one of his heroes (reference to a furore that occurred on publication of Trewavas (1981)) and that the zoological members of his department were laughing about neurobiology in plants. He had hoped that molecular biology would raise their appreciation of plant biology, but this was being diminished by plant neurobiology. I suspect that this is the fundamental reason for Alpi et al. (2007). I refused because I have always believed in open debate.

We do not know the following about the Alpi et al. (2007) petition. The immediate group and outsiders totalled 36 out of how many plant biologists solicited? We have not been told how many contacted but did not sign. The signatories who signed up from outside the immediate group around Taiz and Robinson seemed to have accepted the Robinson statement on trust, suggesting previous contact, friends and similarity of a particular research area played a strong part in those that did sign. Or at least there is no indication that those who signed attempted to investigate the truth of the Robinson claims. That is a serious mistake because there was good justification for rejecting Robinson’s statement whose information seemed to have been acquired second hand.

The original Robinson letter stated that proponents of neurobiology had suggested that higher plants have nerves, synapses, the equivalent of a brain localised somewhere in the root and an intelligence. That was misleading. Darwin was responsible for the statement of the root tip acting not as a brain but “like the brain” of a lower animal. As regards nerves and synapses, there was no mention in Alpi et al. (2007) of JC Bose’s enormous compendium of thousands of experiments and demonstrations of nerve and synapse in *Mimosa* and related sensitive plants and other species too. I suspect that no one in the list of 36 was in any way aware of Bose’s contribution. His publications were in the 1920s. You would have to be in plant electrophysiologist to even have heard of Bose. But his nerves and synapses are functional activities; they behave like nerves and synapses, but the refined and highly differentiated anatomical in animals is not present, and it would not be expected to be so.

To quote Bose, “Experiments are described showing that the response of the isolated plant-nerve is indistinguishable from that of the animal nerve throughout a long series of parallel variation of conditions”. “In the case of responsive plants that exhibit visible movement (like Mimosa or Dionaea) are not unique but occur under similar circumstances even in ordinary apparently insensitive plants and are characteristic of all plant organs” (Bose 1926. p44, p45 and following; Bose 1907, p 15).

I wrote to most of those who signed asking for their reasons. Only four replied; two said it was a spat that would fade quickly, and two others said that they thought it was another version of The Secret Life of Plants (Tompkins and Bird 1973) indicating no clarity of understanding.

Despite intelligence being mentioned in the original letter by Robinson, it is not discussed at all in Alpi et al. (2007). It is not surprising that plant scientists do not read the intelligence literature. I assume like others they considered intelligence, incorrectly, to be the equivalent of human intelligence and IQ. This initial group of 36 in my view signed up for something they had no clear understanding of at all and may have been misled by the compilers of it. It was a false prospectus driven not by the desire to improve scientific knowledge but to condemn. This group of 36 quickly evaporated. Most were evidently not committed and did not join any subsequent venture. It was down to five or six for the arguments about consciousness and is now down to one in this article. Hardly a vote of confidence or perhaps an appreciation that Alpi et al. (2007) got it wrong.

## Conclusion

Disputes in plant science should be about clarifying thought, the dispassionate exchange of ideas and information. I have indicated that debates should be based on reasonable guidelines, and I have indicated some of these. I do think there was an editorial failure in TIPS not to insist on immediate countervailing views so that assessment could be more easily made. But I have put in some guides to a debate which would be helpful if notice is taken of them. The intention behind my introduction in Trewavas (2003) was to upgrade the status of plants indicating that they, like all life, are intelligent. That view has surely had an impact and as an idea is now much more widely spread. The value to me personally is that it drove me to understand better the nature of plant life in the wilderness and taken me into different avenues of understanding which are presently being written up.

Intelligent behaviour is a fundamental capability for all life that exists in the wilderness. Assessments of plant capabilities made solely from laboratory exercises that make plants behave as though they are dogs trained to jump through hoops mislead as to the real complexity of plant behaviour. Consequently, plants can be wrongly described as simple. This is accompanied by further simplistic assumptions that reductionism will explain all. It will not! In wilderness, any individual faces a bewildering array of environmental variation not only in the shoot but from the highly variable nature of the soil that any seed lands on and must accommodate. There is a need for experimental plant scientists to finally eschew the convenience of the laboratory and investigate real plants in real environments.

My aim in introducing plants as intelligent organisms was to improve the public status of plants and that has now occurred. “Are plants intelligent? Of course they are” Castiello (2023). Research on climbing plants easily reveals their intelligent capabilities, and there are some six papers from this group around Castiello (Padua University) in the reference section of this paper that indicate this to be the case. The start here was of course Darwin’s (1882) recognition that climbing plants could recognise the thickness of a potential support and make a decision on whether to use it or not. Intelligent? Of course it is; the probability of survival has increased.
